# Breastfeeding and caring for children: a qualitative exploration of the experiences of mothers with physical impairments in Ghana

**DOI:** 10.1186/s12884-020-03028-1

**Published:** 2020-05-29

**Authors:** Angela Kwartemaa Acheampong, Lydia Aziato, Margaret Marfo, Philomena Amevor

**Affiliations:** 1 School of Nursing, Wisconsin International University College-Ghana, Accra, Ghana; 2 Department of Adult Health, School of Nursing and Midwifery, University of Ghana, Legon, Accra, Ghana

**Keywords:** Discrimination, Disabled mothers, Ghana, Mothering experience, Physical impairments qualitative research

## Abstract

**Background:**

Breastfeeding and caring for children demand time, energy and effort. Mothers with physical impairments in Ghana require special needs to be able to achieve optimal motherhood as society demands. Globally, literature on breastfeeding and caring for children among mothers with physical disabilities is limited. Similarly, there is dearth of literature on the experiences of mothers with physical impairments in Ghana. Therefore, this study sought to add to literature by exploring the experiences of mothers with physical impairments with regards to breastfeeding and how they care for their children.

**Methods:**

Qualitative descriptive exploratory design was used. Twelve mothers with physical impairments who had been purposively sampled gave informed consent before data was collected through in-depth one-on-one interviews. Data was recorded, transcribed and analyzed inductively using the content analysis technique.

**Results:**

Mothers with physical impairments perceived breastfeeding as difficult and expensive due to issues relating to disruption of sleep, dysfunctional limbs and the need for breastfeeding mothers to eat nutritionally balanced meals. Participants felt prejudged and discriminated at different points in their daily encounters.

**Conclusion:**

Mothers with physical impairments have challenges. Therefore, attitudinal change should be advocated in the Ghanaian society with respect to issues concerning people living with disabilities.

## Background

The United Nations Convention on the rights of persons with disabilities stipulates that persons with all types of disabilities are entitled to all human rights including the right to procreate [1]. The World Health Organization (WHO) and the United Nations Population Fund (UNFPA) estimate that, about 10% of the global population live with disabilities [2]. Women in middle income countries have higher prevalence of disabilities compared to those in high income countries [3]. There are 737,743 (representing 3%) persons living with disabilities in Ghana [4]. The total number of adults in the Greater Accra Region of Ghana with disabilities are 103,939 (representing 2.6%) and adult females are slightly more likely to have disabilities than males [5].

Generally, people with disabilities are dependent upon others, and same can be said of women with disabilities. It is the duty of mothers to nurture babies but their efficiency becomes questionable when they are disabled. Consequently, in a systematic review, people doubted the ability of mothers with disabilities to play their roles effectively as parents [6]. Such a classification threatens the self-integrity of people living with physical impairments [7]. But mothers with physical impairments in the United States of America remain central to the nurturing of their babies [8]. In addition to other roles, breastfeeding is de facto for mothers in Ghana. Optimal breastfeeding is a key cost-effective child survival strategy which is beneficial to all infants irrespective of geographical location or cultural background [9].

The WHO recommends that, every infant should be breastfed for a minimum duration of 2 years; 6 months exclusive and 18 months complementary feeding [10]. This is due to the many benefits of breastfeeding to infants which include increase in intellectual capabilities, prevention of allergies, reduction in the rate of childhood obesity and reduction in infant morbidity and mortality [11–15]. Suboptimal breastfeeding is a nutritional disorder and nutritional disorders remain part of the dominant cause of global disease burden [16]. Women with disabilities have a higher probability of giving birth to preterm infants [17, 18]. Pre-term infants have improved cognitive development when they are breastfed optimally with breastmilk [19–21]. Meanwhile, mothers with disabilities have challenges with breastfeeding such as limited support, disability related health considerations, limited information as well as difficulties with milk production and latching which culminate in lower rate of breastfeeding [18, 22]. Mothers with disabilities have a higher risk of developing postpartum depression than mothers without disabilities [17]. Yet, those mothers find it difficult to access reproductive health services since most health facilities do not have the requisite resources to meet their needs [23]. In Ghana, the quality of postnatal healthcare services for women is low [24–26]. Physical environments of most health facilities are such that, they restrict women with disabilities from accessing maternal and child health services [27, 28] coupled with inadequate social support for people living with disabilities [29, 30].

People living with disabilities are discriminated upon in almost all facets of life [31, 32]. According to the United Nations, females living with disabilities are more likely to be discriminated compared to their male counterparts [1]. It was reported in a study conducted in Ghana that, women with physical impairments are physically, socially, sexually and verbally abused [33]. Mothers with disabilities have heightened fears about their abilities to meet the challenges of motherhood due to scepticisms from health personnel who doubt their abilities to be mothers [34, 35]. Some mothers with physical impairments have reported both physical and mental challenges [36]. In a systematic review of literature from different parts of the globe, it became evident that, mothers with disabilities have limited access to reproductive health services [37].

This study was situated in the philosophical foundation of the social model of disability by Mike Oliver. The social model of disability states that disability is defined by the way the society is organized rather than a person’s physical impairments [38]. Thus the fundamental assumption of that model is that, ‘disability’, as constructed by this model is a consequence of social and multiple other societal barriers. The social model of disability has been used to study the daily lives of persons with disabilities across the globe [39–43]. The model can be related to the way a society upholds the rights of persons with disabilities. In the Ghanaian cultural context, people with disabilities are mostly deemed as helpless and unable to achieve anything meaningful. Therefore, they are mostly not recognized, disrespected and discriminated against. This notion of the Ghanaian populace makes people living with disabilities feel more incompetent than they really are. The United Nations Convention on the Rights of Persons with Disabilities (UNCRPD) draws on the social model of disability which was signed and ratified by Ghana in 2007 and 2012 respectively. There is paucity of literature on the experiences of mothers with physical impairments in the Ghanaian context with respect to breastfeeding and how they care for their children. This study seeks to fill part of this gap in knowledge by exploring the experiences of mothers with physical impairments in the Greater Accra Region of Ghana. In this study, disability is referred to as any physical impairment of the limbs of mothers which causes inefficient use of such limbs, hence meddling with their day to day activities.

## Methods

### Aim, design and setting

This study employed qualitative descriptive exploratory design to explore the experiences of mothers with physical impairments with respect to breastfeeding and caring for their children, in the Accra Metropolis of Ghana. Therefore, the research question was ‘what are the experiences of mothers with physical impairments with respect to breastfeeding and caring for their children?’ This qualitative approach allowed detailed descriptions of participants’ life experiences and the meanings they attach to such experiences [44]. The study was conducted in Accra, the capital city of Ghana which is home to about 16% of the total population of Ghana. The Greater Accra Region is made up of people from all parts of the country. Interviews took place at a rehabilitation centre for people living with disabilities in Accra, Ghana. The rehabilitation centre is resourced with equipment which are used in training persons living with disabilities to learn a trade. The centre is also the venue for monthly meetings of persons living with disabilities. Since it is the central meeting place of persons with disabilities we accessed participants from different parts of Ghana. Thus the context of the study attracted mothers with physical impairments from all parts of Ghana.

### Sampling and data collection procedures

Twelve mothers with physical impairments were interviewed individually after they had been selectively sampled by the researchers based on the inclusion criteria. The inclusion criteria were: Ghanaian women with physical impairments (difficulty in the use of their limbs) who had biologically birthed children within the last 10 years, breastfed them and personally cared for them. They were also the ones who voluntarily opted to participate in the study. Data collection and analysis were done concurrently. Participants were approached by researchers through a staff of the rehab during one of their monthly meetings after permission had been sought from the association of persons with disabilities. The objectives and eligibility criteria for participating in the study were explained to them. Those who opted to participate were given consent forms for them to provide written consent. Participants were contacted later to meet the researchers at the rehabilitation centre on days and times that were convenient for them to be interviewed. The interviews were conducted in English and Twi (the most popular Ghanaian language) by the first and second authors who are fluent in both languages. A semi-structured interview guide was used to elicit responses from participants. The questions were the ones that could allow in-depth probing of emerging themes. For instance participants were asked: *Can you describe your experiences with breastfeeding as a mother*? *Tell me about your experiences as you care for your child. Can you describe the challenges you have faced so far as a mother with physical impairments?* Participants were allowed to express themselves freely without interferences during the interviews and leading questions were avoided to ensure that participants’ experiences remained intact. Participants were interviewed individually and each interview lasted between 30 and 40 min. Data reached saturation on the twelfth participant because at this point, no new information was obtained from participants. Interviews were audiotaped and transcribed verbatim into English. Transcripts from Twi interviews were discussed with experts in the Twi language to ensure that participants were faithfully represented.

### Data analysis

Data were analyzed according to the processes involved in content analysis and as described by Padgett [45]. Manual processes described were not used because the NVivo software was used to manage the data. Content analysis was the analysis of choice because there were no pre-meditated themes that guided the study. Transcripts were read several times by reviewers to make meaning of participants’ narrations. Transcripts were coded by reading individual sentences critically and allocating words or phrases that captured the meanings of the sentences. Similar codes were grouped together to form sub-themes while sub-themes were grouped together to form major themes. To ensure that the data were well represented, the research team met and thoroughly discussed the themes and sub-themes. The data were then imported into NVivo, version 11 for management. The study held that, the data reflected the participants’ experiences in respect to breastfeeding and caring for a child as physically disabled women.

### Trustworthiness of the study

Interview guide was developed based on the objectives of the study and it was pilot tested with four mothers with similar characteristics as the participants. Corrections were made after that by the authors before it was used for data collection. Concurrent data collection and analysis ensured that, emerging themes were further probed in subsequent interviews to get other experiences and to fully understand the context of the data. The same interview guide was used to interview all participants to ensure consistencies. Field notes were taken and observations were noted to help in triangulation of data. The research team discussed the themes and sub-themes to ensure that participants’ experiences were faithfully presented. Direct verbatim quotes were used to support the women’s experiences to provide context and voice to the women.

### Ethical considerations

Ethical clearance was sought from the Institutional Review Board of the Noguchi Memorial Institute for Medical Research (NMIMR-IRB CPN 038/16–17, FWA 00001824). A letter was sent to the Ghana Federation of Disability Organizations for permission to interview their members. All participants gave informed consent to participate in the study and they were made to understand that, participation in the study was voluntary and that, anyone could withdraw from the study at any time without any consequences. Identification codes were used to represent the participants to ensure anonymity.

## Results

### Participants’ background

Twelve participants were recruited into the study. They were aged between 21 and 57 years old. Ten of them were Christians while two were Muslims. Seven of the participants were living with their spouses whereas five of them were single. All the women who participated in the study had biological children aged between 1 month and 10 years. Five of the women had a child each while the other five had two children each. Each of the last two mothers had three children. Although all participants were residents of the Greater Accra Region but originated from different parts of Ghana. Six of the women were unemployed and the rest were employed in the informal sector. Six of the mothers had basic education, two had secondary education and the rest had no formal education.

There were two broad themes which emerged; breastfeeding experiences and challenges surrounding child care. Sub-themes which emerged after data analysis included; decision to breastfeed, breastfeeding emotions, mobility, access to buildings, finances, prejudice and discrimination.

### Breastfeeding experiences

This theme describes the breastfeeding experiences of mothers with physical impairments. Under breastfeeding experiences, the women described sub-themes which were related to their decision to breastfeed and emotions which emerge as a result of breastfeeding. These have been described with verbatim supporting quotes.

### Decision to breastfeed or otherwise

Some of the participants practiced breastfeeding because of the influence of their mothers. It was either their mothers who told them to breastfeed or they observed their mothers’ breastfeeding.

***DM11****: ‘ … . I came and met my mother in this world and she told me that she practiced breastfeeding so based on that, I also decided to practice breastfeeding when I gave birth. My mother is my role model and she was on earth before I came so whatever she says is very important to me’.****DM12****: ‘What my mother asked me to do was what I did after I gave birth. If my mother had asked me to practice exclusive breastfeeding I would have done it’.*

Some also decided to breastfeed because of their admiration towards breastfeeding mothers.

***DM4****: ‘ … When I see someone after delivery and she is breastfeeding, I do admire it so that was what made me decide to breastfeed after delivery’.****DM3****: ‘before, when I had not delivered, I used to really admire it. So, I made up my mind that, me too when I deliver I will breastfeed my child’.*

Others decided to breastfeed because of the counsel they received from their nurses and their personal convictions about breastfeeding. Some reported that, the love they have for their children, the naturalness of breastmilk and the fact that it was created by God were what influenced their decisions to breastfeed.

***DM9****: ‘I love my children and therefore I want the best for them. The nurses told me that when the children are breastfed, they become intelligent and they grow well as well. So I decided to breastfeed so that my children would be strong and intelligent’.****DM8****: ‘Yes when I went to the hospital, the nurses told me that I should practice exclusive breastfeeding for six months without any water so I did’.****DM10****: ‘I had to breastfeed my baby because it has been there since life itself began and it is natural. Breastmilk is something which was created by God for children as food’.*

### Emotions emerging from breastfeeding

Breastfeeding evoked emotions such as joy, love anger and pain in some of the women. Breastfeeding was considered as the source of love between mothers and their children. Some felt annoyed for not lactating sufficiently and others enjoyed the act of breastfeeding.

***DM3****: ‘I was annoyed that some people could breastfeed their children but for me I could not breastfeed to my satisfaction because I could not produce enough breastmilk’.****DM4****–‘It’s because it is good. It is very good that persons with disability are also able to enjoy breastfeeding and when I got my child, I also enjoyed the art of giving breast milk’.****DM5****: ‘Breastfeeding allowed me to be able to love my child extraordinarily, the more I breastfeed and the child also looks into my face, the more the love between us becomes strong. Whilst giving my child breast milk, she looks into my face and touches my breast, she develops some love and bond for me and the same way … ’*

Some mothers narrated that they felt pains anytime they sat to breastfeed and had to find other means to breastfeed successfully such as sleeping by the baby to breastfeed, feeding at short intervals and lying flat beside the baby to breastfeed.

***DM4:****‘ … I have to always sleep by him to be able to breastfeed. I feel pains in my waist when I sit down so I cannot breastfeed when I sit. I cannot breastfeed my baby when I cannot find a space where I can lie beside the baby’.****DM2:****‘me too when I sit down for too long to breastfeed, my back hurts. I have to breastfeed for shorter intervals. After every five minutes, I have to stop and relax before continuing to breastfeed’.****DM8****: ‘During the early days of breastfeeding, apart from the pain I felt in my nipple, I always felt back pain’*

### Challenges pertaining to child care

Mothers described the challenges they faced on a daily basis as they cared for their children. Majority of the mothers needed assistance to attend to their daily activities and to care for their children although seven of them were living with their spouses. Others were also independent and could navigate through their daily activities without the need for assistance. Sub-themes included; access to buildings, finances, mobility, prejudice and discrimination.

### Access to buildings

Most mothers reported that, they had limited access to both public and private buildings. Some of them had to be carried into public buildings because they could not access such buildings due to lack of ramps and other structures which could make it easy for them to access such facilities.

***DM1****: ‘hmm, the church I attend, I have a problem so now I don’t go to church, they have built it in such a way that there are steps and if I get there, I need people to carry me before I can enter the building and if we close they are supposed to carry me down, … because they carry me I don’t feel happy to go so for four or five months, I have not gone to church’****DM4****- ‘You see … . My landlord has dug a manhole in front of my room. This makes it difficult to carry my child around the house … they don’t think about where I will pass to go into my room. They do it, just do it. They don’t consider that I even have a child or that I am a person with disability. They don’t consider that I am also a human being’.****DM8****: ‘ … there are no ramps, the building has steps. Therefore, when I have to climb the steps, I suffer especially when my child is with me. It becomes very difficult to carry my child around … ’*

### Finances

Most of the mothers faced financial challenges. Some struggled to make ends meet and meet the demands of their children. This emanated from sources such as rejection by spouses, inability to secure jobs and inadequate professional training.

***DM1****: ‘ … sometimes you the mother, you have to eat so that you get breast milk to feed the child, may be by that time, I will not have money because I can’t secure any formal job and the father too has not brought money for me to buy those things’.****DM12****: ‘I have financial problems. I had to relocate with my son from this area because of my finances’.****DM7****: ‘ … I don’t have anyone to help me financially. I have learnt how to sew before, but then I was unable to rent a store, … nobody too is helping me financially to take care of my child. So even with the dressmaking, the skill has left me … . like I am not even the one who learnt how to sew. Currently, I cannot even … sew’*

### Mobility

The mothers described different ways of moving from one point to the other as they go about their daily livelihoods. Some crawled with their babies, others sat in wheel chairs with their babies on their laps and others also used crutches and calipers to remain mobile.

***DM8****: ‘I carry my child at my back and hold her in place with a cloth anytime I have to descend stairs with her. Once the baby is tied to my back, I use my crutches and calipers to go anywhere with her without any form of assistance’.****DM11****: ‘I am unable to walk. I use crutches, caliper and wheelchair to be able to move around and go about my motherly duties. I mostly put the baby on my laps and sit in my wheelchair before I propel it manually to get to my destination’.****DM5****: ‘up to today. I have been crawling on the floor for some time. Even at home, for me do attend to my daily activities as fast as possible, I crawl on the floor to attend to my child’s needs. I only carry my baby on my laps and use my wheelchair when I have to go out’.*

There were participants who had to crawl on the floor at night to reach their babies whenever they cried to be able to breastfeed.

***DM6****: ‘ … when my baby cries for breastmilk, I have to crawl on the ground in order to meet her breastfeeding demands’****DM7:****‘I crawl on the floor to pick my baby and breastfeed anytime he demands for breastmilk’.****DM4****: ‘ … when she’s crying I have to crawl to where she is and carry her on my own and breastfeed’.*

Some of the women narrated that they felt pain in their legs when walking and that causes them to sometimes fall without warning. This puts mother and baby in danger and causes a lot of embarrassment and casualties.

***DM9****‘Although today, the pain is bearable, when I walk a little, I feel a lot of pains in my leg and that causes me to fall at any time. I fall down without any warning all the time. Sometimes, when I am carrying my child, I fall down suddenly with her without any warning. Sometimes, I fall down at public places. This really causes a lot of embarrassment for me’.*

Some women perceived breastfeeding to be expensive due to the cost involved in transportation since the baby would always have to tag along during the exclusive breastfeeding stage. Restricted mobility in some circumstances meant that, some mothers had to always charter taxies before they could move round with their children.

***DM9****: ‘I perceive breastfeeding to be expensive. Because the baby had to tag along with me all the time, I was forced to charter taxis all the time which had a strain on my finances. … whenever I didn’t have money to charter a taxi, I could not send my baby for child welfare clinic. This was because, carrying the baby and walking caused a lot of pain to me’.*

### Prejudice

The women felt judged by the general population right from the ante-natal period to the time they became mothers. They perceived that most people thought they should not be mothers because of their physical impairments. To them, most people were skeptical about their abilities to be competent mothers. One woman reported that, people used to stare at her baby bump and that made her embarrassed to the point of crying.

***DM4****: ‘ … .it is small. When I gave birth, there was this woman in our house she used to laugh at me. She used to say “But this one if she gives birth what breastmilk will the baby suck?” They’ll make jokes about my ability to be a mother and they will be laughing at me.****DM3****: ‘Once, I was carrying my child when I stopped by someone to ask for direction. The moment he saw us, he ignored us and refused to even talk to me. I then told him that I am looking for direction. Then he said “Oh, sorry I thought you were here to beg for food and money for your child”. … I don’t blame them because the perception is that, those of us mothers with physical impairments cannot be competent mothers’.****DM9****: ‘When you are a woman with physical disability and pregnant, people actually take their time to stare at you when you get obviously pregnant. Those people can stare to an extent that, you sometimes feel extremely embarrassed. My situation too is such that, I easily fall down uncontrollably. Therefore, when people begin to stare at me, I feel extremely embarrassed and that makes me fall down frequently. Sometimes I cry my head out because of the way people stare at me and judge me*’

### Discrimination

The mothers gave different accounts of situations whereby they felt discriminated upon. Sometimes, the discrimination occurs as a result of the perception by some members of the community that physical disability is contagious. In most of their encounters with different people, they felt discriminated. One of them cried anytime she felt discriminated.

***DM6****: ‘Sometimes, in performing my duties as a mother, people discriminate against me. When I take my baby for “weighing” (child welfare clinic), the other mothers behave as if I am contagious. A lot of the others refuse to sit close to me. They all move to the other chairs and squeeze themselves on the other chairs while my chair remains empty’.****DM7****: ‘ … Right now, when I carry my baby at my back through town, people stare at me as if I am strange. They make funny comments like; “how can a sick person carry a child at her back?” This is how come I don’t carry my child in public again. When I have to carry her, I do it in the confines of my home’****DM9: ‘****Once, at the house, my child strayed into the street and a stranger brought her inside. When he came, he met about five of us who were women in the house, he went round asking all the women if they were the mother of my child without asking me. It took the other women to convince him before he believed that I was the mother of the child... Most people discriminate against me; … . I cry a lot most of the time due to the way people discriminate against me’.*

## Discussion

This study probed into the experiences of mothers with physical impairments in the Greater Accra region. Breastfeeding practices among mothers with physical impairments were varied. Some mothers felt pain during breastfeeding. Nipple and breast pain during breastfeeding has been reported in the literature [46, 47]. Inadequate positioning can cause pain during breastfeeding [48] and the effect of breastfeeding pain on the mother can have negative consequences [49]. Therefore nurses should educate such mothers on the best way to position the baby during breastfeeding. Mothers who had to crawl in order to reach their babies possibly had no assistance and had to find their own means of satisfying the breastfeeding demands of their babies. Assistive mobility devices should be within easy reach of mothers always.

Mothers with physical impairments perceived breastfeeding as expensive although breastfeeding is viewed as cheap among mothers practicing formula feeding [50]. Such perceptions can have negative outcomes on breastfeeding. This emphasizes the need for breastfeeding counselling for mothers with physical impairments. Therefore, breastfeeding counselling should be introduced early and regularly to mothers with physical impairments for them to have positive perceptions about breastfeeding to improve breastfeeding outcomes.

The decisions of the mothers to breastfeed were influenced by admiration for the act of breastfeeding, love for their children, advice from their mothers, breastfeeding beliefs and education received from nurses about breastfeeding. It has been documented that the opinions of family members and healthcare providers are predictors for the breastfeeding decisions of mothers [51]. In other studies, factors which influence mothers’ breastfeeding decisions included antenatal depression [52], cultural differences [52], mothers’ health beliefs [53] and the opinions of mothers of breastfeeding women [54]. The different groups of mothers and the locations of the studies reiterate the fact that mothers in general go through decision making processes on the issue of breastfeeding. Therefore, the decision making process of mothers with physical impairments may be improved if factors identified are considered during breastfeeding counselling to improve breastfeeding initiation and continuation.

The mothers’ generally viewed breastfeeding as an activity which proved their abilities to also be mothers since some people such as neighbours and the general public doubted their abilities to perform their roles effectively as mothers [35]. Therefore, most of them enjoyed the art of breastfeeding. This appetite to breastfeed could be as a result of the general notion among the Ghanaian cultural context that people with disabilities were incapable of performing activities just like their counterparts without disabilities. One of them became annoyed because she could not breastfeed to her satisfaction due to scanty milk production. The art of breastfeeding evoked love between some mothers and their children. Breastfeeding should be encouraged among mothers since it has been documented to increase the bond and cognitive abilities of children [55].

Participants went about their daily activities as mothers caring for their children with challenges. The challenges narrated by mothers with spouses were not so different from mothers without spouses. This may be due to the patriarchal nature of the Ghanaian society which promotes childcare as women’s role. The challenges could partly be linked to the infringement on the rights of people living with disability which is determined by the way society is molded according to the social model of disability. Meanwhile, Ghana is signatory to the UNCRPD. Therefore, the onus lies on the society to make the lives of mothers living with physical impairments better.

The mothers narrated difficulties in accessing buildings during pregnancy and after delivery when they had to access certain buildings with their children. This may be due to the numerous infrastructure in Ghana which have not been built with features that makes it accessible to persons with disabilities. This resonates with a study whereby mothers with physical impairments had difficulties navigating buildings [37]. Meanwhile, the immediate environment of persons with disabilities has effect on their social interactions and participation in activities [56]. Government should enforce the accessibility to buildings law to ensure that people living with disabilities get access to buildings. This would make it easy for mothers with physical impairments to meet the demands of motherhood.

Mothers lamented about their inability to make ends meet due to financial challenges as a direct effect of their inability to secure stable jobs. Perhaps employers consider mothers with physical impairments as incompetent and that may explain the reason for their inability to secure stable jobs. The mothers’ narrations are congruent with an experimental study where employers refused to employ applicants who pretended to be disabled [57]. People with disability complain of discrimination by employers most of the time [58]. Laws should be enforced for corporate bodies to employ the stipulated quota of staff who fall within the disability group. This may ameliorate the issue of unemployment among this category of mothers who have to be financially stable in order to meet the needs of their mothers.

The use of assistive devices presents with problems related to acquisition, affordability, repair and maintenance of such devices [59]. Some mothers narrated that they use assistive devices to be able to move around with their children. One of them reported that she had to always crawl to attend to her daily activities as a mother. In the Ghanaian context, most mothers have to carry their children around in spite of the bad roads. This may pose serious problems for mothers with physical disabilities who have to carry their children around. This suggests that mothers with physical impairments have peculiar mobility restrictions and therefore, there is the need for assistance for them to acquire assistive devices in order to meet the demands of motherhood. Therefore, people with physical impairments should be given mobility devices as part of the national health insurance benefits for them to be mobile.

Most of the mothers narrated instances whereby they were prejudged and looked upon as incompetent to perform their roles as mothers and be responsible. Some mothers were automatically considered as beggars by the public and therefore, they were shunned. Others were also considered as incompetent to be mothers and not to talk of breastfeeding. Perhaps the notion that disabled people are automatically dependent is what drives this attitude of prejudice. The public then assumes that it is impossible for such mothers to perform their motherly roles. Prejudice should be discouraged among the general public through education since chronic stereotyping can have negative impact on the self-integrity of people with physical impairments [7].

Mothers reported their encounters that made them feel discriminated against. Discrimination of persons with disabilities has been reported in the literature [60–63]. Women with disabilities are socioeconomically disadvantaged compared to their male counterparts [60]. The mothers’ account suggested that, members of the community treat them as if their conditions are contagious. This may be due to lack of education on the nature of physical impairments. Health professionals should educate the public about the causes of physical impairments to avert such situations. The whole society’s way of relating to persons with disabilities should be refined through enactment and implementation of strategies that would let the UNCRPD come to light. The social model of disability should also be used as a guide by health professionals to educate the public on issues relating to disabilities.

The study did not include the experiences of mothers with other forms of disabilities such as intellectual disabilities. Thus this limits the application of this findings to mothers with other forms of disabilities. The sample size of the study does not allow generalization of the findings.

## Conclusion

It can be inferred that, breastfeeding and caring for infants gave a sense of hope to the participants. In as much as some of the participants enjoyed the nurturing role of mothering their infants, most of them faced an array of problems as they performed their roles as mothers. Challenges ranged from discrimination and prejudice, to difficulties in accessing buildings and securing stable jobs. Such challenges impeded their desire to meet the demands of motherhood. Consequently, portions of the UNCRPD that touch on the reproductive rights of women with disabilities should be implemented and enforced to ease some of the challenges faced by women with disabilities in Ghana. Laws concerning accessibility to buildings should be enforced. Assistive devices should also be made readily available to mothers with disabilities to ease mobility for them. It is also recommended that, attitudinal change should be advocated in the Ghanaian society with respect to issues concerning people living with disabilities.

## Limitations

The authors limited the study participants to only breastfeeding women with disabilities. Those with disabilities who did not breastfeed would have had a different perspective.

## Supplementary information

**Additional file 1.**

## Data Availability

The data sets analyzed during the current study is available from the corresponding author on reasonable request.

## Abbreviations

WHO: World Health Organization
UNFPA: United Nations Population Fund
UNCRPD: United Nations Convention on the Rights of Persons with Disabilities
