# Supplementary material for: Breastfeeding and caring for children: a qualitative exploration of the experiences of mothers with physical impairments in Ghana
Source: BMC Pregnancy Childbirth. 2020 May 29;20:331. doi: 10.1186/s12884-020-03028-1 (PMC7260762; doi:10.1186/s12884-020-03028-1)
Supplement: Supplementary file 1 — Additional file 1. [file 12884_2020_3028_MOESM1_ESM.docx]

**DATA COLLECTION INSTRUMENT**

**INTERVIEW GUIDE FOR BREASTFEEDING MOTHERS WITH PHYSICAL DISABILITIES**

**SECTION A: Demographic Information (Background Information)**

ID number---------------

Age: 18 – 25 26 – 30 31 -35 36 – 40 41 – 45  46 – 50 Above 51

Religion ------------------------------------------------------------------------------------------

Marital status-------------------------------------------------------------------------------------

Number of children (biological) --------------------------------------------------------------

Age(s) of children ------------------------------------------------------------------------------

Place of Residence -----------------------------------------------------------------------------

Region of Origination -------------------------------------------------------------------------

Ethnicity -----------------------------------------------------------------------------------------

Employment Status ----------------------------------------------------------------------------

Level of Education -----------------------------------------------------------------------------

Nationality---------------------------------------------------------------------------------------

**SECTION B: Guiding Questions**

**Breastfeeding experience of mothers with disabilities**

Can you describe your experiences with breastfeeding as a mother?

- How long have you breastfed and why?
- When do you intend to stop breastfeeding this child and why?
- How often (day/night) do you breastfeed?
- Hourly
- On demand
- If it is on demand, describe the signal by your baby that tells you to breastfeed

**Factors that influenced the decision to initiate breastfeeding**

What informed your decision to choose to breastfeed?

- Previous experience
- Experience of other mothers
- Midwives’ initiation
- What encourages you to breastfeed your infant?
- Health benefits
- Affordability
- Norm or cultural expectation

**Psychological reaction and emotions relating to breastfeeding**

- What are your thoughts about the demands of breastfeeding?
- How easy or difficult is it?
- Describe the emotions you experience anytime you breastfeed your child.

**Challenges pertaining to childcare**

Tell me about your experiences as you care for your child

How do you manage to keep up with the demands of your baby?

- Time management
- Interrupted sleep
- How has this baby affected your way of life in keeping up with activities of daily living as well as your relationship with others?
- Your ability to be mobile
- The way people relate to you
- Can you describe the challenges you have faced so far as a mother with physical impairments?
